# Accurate GFR in obesity—protocol for a systematic review

**DOI:** 10.1186/s13643-019-1052-2

**Published:** 2019-06-22

**Authors:** Sriram Sriperumbuduri, Robert Dent, Janine Malcolm, Swapnil Hiremath, Ran Klein, Christine A. White, Pierre Antoine Brown, Ayub Akbari

**Affiliations:** 1 Department of Medicine, Division of Nephrology, The Ottawa Hospital, Ottawa, Canada; 2 Department of Medicine, Division of Endocrinology, University of Ottawa, Ottawa, Canada; 3 Kidney Research Centre, Division of Nephrology, Department of Medicine, The Ottawa Hospital, University of Ottawa, Ottawa, ON K1H 7W9 Canada; 4 Department of Medicine, Division of Nuclear Medicine, University of Ottawa, Ottawa, Canada; 5 Department of Medicine, Division of Nephrology, Queen’s University, Kingston, Canada

**Keywords:** Obesity, eGFR, CKD, Systematic review, Meta-analysis

## Abstract

**Background:**

Obesity is increasing globally. Chronic kidney disease (CKD) is strongly associated with obesity. Kidney function is commonly estimated with equations using creatinine (such as CKD-EPI equation) which is a product of muscle metabolism. Decisions about categorizing CKD, planning modality of renal replacement therapies, and adjusting dosages of medications excreted by the kidneys are done using these equations. However, it is not well appreciated that creatinine-based equations may not accurately estimate kidney function in obese individuals. We plan a systematic review of diagnostic studies which will compare estimating equations to actual measured kidney function.

**Methods:**

We will systematically search electronic bibliographic databases including MEDLINE, EMBASE, and the Cochrane Library with no restrictions on language or specific dates. The search terms will be adapted for the different databases using a combination of Medical Subject Heading and relevant keywords contained in titles and abstracts. Our preliminary search strategy using Cochrane, MEDLINE, and EMBASE databases have identified 190, 1246, and 1660 citations, respectively. For all studies selected, we will extract information on general study characteristics, study participant (age, sex, ethnicity, weight, height, BMI, BSA), type and protocol of reference standard utilized, the index test studied, the methodology of measurement of index test, categories of GFR, the proportion of eGFR within 10, 20, 30, 40, and 50% of measured GFR, and bias between eGFR and measured GFR. If the quality of methods and risk of bias are adequate, we will perform a meta-analysis. We will assess the heterogeneity using the *χ*^2^ and the *I*^2^ statistics to examine whether the estimates from studies included could be pooled. Sensitivity and multivariate meta-regression analyses will be performed to assess the effects of clinical factors and socio-demographic characteristics reported in included studies on the meta-analytic estimates. All analysis will be performed using the Comprehensive Meta-analysis software.

**Discussion:**

This systematic review might help to inform clinicians on the best equation to use in patients with obesity and CKD for staging of CKD and for medication dosing. If no equation is deemed suitable, this review will form a basis for future studies of GFR in obese individuals.

**Systematic review registration:**

PROSPERO CRD42018104345

**Electronic supplementary material:**

The online version of this article (10.1186/s13643-019-1052-2) contains supplementary material, which is available to authorized users.

## Background

Obesity is defined on the basis of body mass index (BMI) ≥ 30 kg/m^2^ and divided into 3 classes (class 1, BMI ≥ 30 and < 35; class 2, BMI ≥ 35 and < 40; class 3, BMI ≥ 40) [1]. It is a global health issue and its prevalence is estimated to increase markedly. The prevalence of obesity is estimated at 13% worldwide. In Canada, the prevalence of obesity in adults was much higher at around 21%, and in the USA, around 30% [2] as of 2012. In a report from Canadian Institute for Health Information (CIHI), the prevalence of class 1, 2, and 3 obesity was 17.4%, 4.6%, and 2.2% respectively for males and 12.7%,7.15%, and 3.8% respectively for females{https://secure.cihi.ca/estore/productFamily.htm?locale=en&pf=PFC1636, #183}.

Obesity is associated with high mortality and morbidity including a higher incidence of cardiovascular disease, hypertension, malignancy, diabetes, and chronic kidney disease (CKD) [1, 3–9]. Most patients with type 2 diabetes have obesity [10, 11] and type 2 diabetes is the leading cause of end-stage kidney disease {CIHI, 2015 #177}. Apart from diabetes itself, obesity is also independently associated with CKD [7, 12]. To facilitate prognostication and management of CKD, it is classified into 6 glomerular filtration rate (GFR) categories (formerly known as stages) [GFR category (stage) 1: GFR ≥ 90 ml/min/1.73 m^2^, GFR category (stage) 2: GFR ≥ 60 and < 90 ml/min/1.73 m^2^, GFR category (stage) 3a: GFR 45 to < 60 ml/min/1.73 m^2^, GFR category (stage 3b): GFR ≥ 30 and < 45 ml/min/1.73 m^2^, GFR category (stage) 4: GFR ≥ 15 and < 30 ml/min/1.73 m^2^, and GFR category (stage) 5: GFR < 15 ml/min/1.73 m^2^] [13].Many commonly used medications are excreted by the kidneys and need dose adjustment which is also based on GFR [14].

Accurate measurement of GFR is cumbersome. It involves injecting exogenous substances (examples: inulin, iothalamate, iohexol, radioactive ethylenediaminetetraacetic acid [^51^Cr EDTA], radioactive diethylene-triamine-pentaacetic acid [^99m^Tc DTPA]) and measuring levels of these substances in the blood and/or urine over a period of time and performing calculations. These methods are all time consuming, logistically challenging, not widely available, and expensive [15]. Canadian and international guidelines (the Kidney Disease Improving Global Outcomes [KDIGO]) recommend the use of estimated glomerular filtration rate (eGFR) for categorization (staging) of CKD and for medication dosing [13, 16]. There are various equations that can be utilized to calculate eGFR (Table 1) all of which utilize endogenously produced small molecules (creatinine, cystatin C, or beta-trace protein [BTP]) combined with certain demographic factors as shown in Table 1. Only one equation was developed in obesity [17, 16] but is not in common use.

**Table 1:** Showing the various eGFR equations used in CKD population

Equation name	Equation	Mean BMI in derivation cohort (SD)
^20^CKD-EPI _creatinine_	eGFR = 141 ×min(Scr/*κ*, 1)^*α*^ × max(Scr/*κ*, 1)^−1.209^ X 0.993^Age^ × 1.018 [if female] × 1.159 [if black] where:	28 (6)
	*S*_cr_ is serum creatinine in μmol/L,	
	*κ* is 61.9 for females and 79.6 for males,	
	*α* is − 0.329 for females and − 0.411 for males,	
	min indicates the minimum of *S*_cr_/*κ* or 1,	
	and max indicates the maximum of *S*_cr_/*κ* or 1.	
	The unit of results is ml/min/1.73 m^2^	
^34^CKD-EPI _Cystatin C_	eGFR = 133 ×min(*S*_cys_/0.8, 1)^−0.499^ × max(*S*_cys_/0.8, 1)^−1.328^ × 0.996^Age^0.932 [If female]	28 (6)
	The unit of results is ml/min/1.73 m^2^	
^42^CKD-EPI _BTP*_	eGFR = 55 *^BTP−0.695^ × 0.998^Age^ × 0.899 [If female]	30.1 (6.4)
	The unit of results is ml/min/1.73 m^2^	
^25^Cockcroft-Gault	Creatinine clearance (eGFR) = ((1.23 [If male], 1.04 [If female) × Weight in kg × (140 − age))/Creatinine	Not reported
	The unit of results is ml/min	
^34^CKD-EPI _creatinine-cystatin C_	eGFR = 135 ×min(*S*_Cr_/*κ*, 1)^α^ ×max(*S*_Cr_/*κ*, 1)^−0.601^ × min(*S*_cys_/0.8, 1)^−0.375^ × max(*S*_cys_/0.8, 1)^−0.711^ × 0.995^Age^ × 0.969 [if female] × 1.08 [if black]	28 (6)
	*κ* = 61.9 (females) or 79.6 (males)	
	*α* = − 0.248 (females) or − 0.207 (males)	
	min(*S*_Cr_/*κ* or 1) = indicates the minimum of *S*_Cr_/*κ* or 1	
	max(*S*_Cr_/*κ* or 1) = indicates the maximum of *S*_Cr_/*κ* or 1	
	min(*S*_cys_/0.8, 1) = indicates the minimum of *S*_cys_/0.8, 1	
	max(*S*_cys_/0.8, 1) = indicates the maximum of *S*_cys_/0.8, 1	
	The unit of results is ml/min/1.73 m^2^	
^53^Jelliffe	Creatinine clearance (eGFR) = 98–16×(age − 20/20)/(Creatinine/88.4). For females, multiply by 0.9	Not mentioned
	The unit of results is ml/min/1.73 m^2^	
^19^Salazar-Corcoran	For males creatinine clearance (eGFR) = (137 − age)× ((0.287 ×weight in kg) + (12.1×height in meters^2^))/51×Creatinine	Developed for obesity but not in common use
	For females creatinine clearance (eGFR) = (146 − age)× ((0.287 ×weight in kg) + (9.74 ×height in meters^2^))/60×Creatinine	
	The unit of results is ml/min	

CKD categorizing depends on GFR, with thresholds at 15, 30, 45, 60, and 90. Accurate assessment of GFR in obese individuals is of paramount importance for categorizing (staging) of CKD and is important at a public health level and an individual patient level. For example, current estimates of the prevalence of CKD categories in Canada are 9.4% for category (stage) 1 or 2 and 3.1% for categories (stage) 3–5 [18]. Incorrect estimation of GFR will lead to incorrect categorization (staging) of CKD which has a major impact on public health statistics and in turn in the allocation of resources. Incorrect categorization (staging) of CKD would also lead to incorrect treatment targets leading to under or over treatment in obese individuals with CKD.

The preferred treatment of end-stage renal disease is kidney transplantation. Kidney transplantation before initiation of dialysis, i.e., pre-emptive transplantation, usually with a live donor, has a better prognosis, and is encouraged when possible. Kidney transplantation is indicated in progressive kidney disease when GFR is < 15 ml/min/1.73m^2^ [19]. Pre-emptive transplantation requires a few months of preparation to work up the potential recipient-donor pair, and prognostication depends on progression and level of kidney function, done with the help of eGFR. Thus, inaccurate assessment of GFR may have a profound impact on timely pre-emptive kidney transplantation.

The preferred (optimal) method of dialysis initiation is either with a fistula (hemodialysis) or a peritoneal dialysis catheter (peritoneal dialysis). Planning for the creation of both fistula and peritoneal dialysis catheter insertion needs to be started in advance of patients needing dialysis. This includes the decision-making, referral to the surgical team, access creation, and healing/maturation to allow dialysis initiation with an optimal/permanent access. Such a process can take up to months in advance of starting dialysis, and eGFR levels and trajectory are used to project and plan access creation (https://www.niddk.nih.gov/health-information/kidney-disease/kidney-failure?dkrd=hispt1319). Thus, incorrect estimation of GFR may lead to obese patients starting dialysis sub-optimally. Indeed, we have shown that each unit increase in BMI increases the risk of sub-optimal dialysis start by 7% [20].

Dosing of renally excreted medications depends on an accurate assessment of GFR. Commonly dosed medications for which this is important include many antibiotics as well as many anti-diabetic medications. For example, metformin (a cheap antihyperglycemic agent) not only controls blood sugar but also helps patients in losing weight [21–25]. SGLT2 inhibitors (canagliflozin, dapagliflozin, empagliflozin) have shown to retard the progression of kidney disease, decrease cardiovascular morbidity and mortality, and help with blood pressure reduction and weight loss [26–28]. Metformin is contraindicated with GFR < 30 and SGLT2 inhibitors should not be initiated if GFR is < 60 ml/min/1.73m^2^. Overestimation of GFR in the obese CKD patient would lead to the use of medications that are contra-indicated, or use of medications at higher doses, leading to higher rates of adverse events (e.g., lactic acidosis with metformin) or even lack of efficacy (e.g., SGLT2 inhibitors). On the other hand, underestimation of GFR in the obese CKD patient would lead to unnecessary avoidance of medications which could be quite beneficial in obese individuals and perhaps an earlier shift to the use of insulin. Similarly, for antibiotics, under-dosing of medication could result in an undertreated infection, contributing to prolonged illness and rise of antibiotic resistance, whereas over-dosing may lead to toxicity. Since obesity is common and associated with many major illnesses, inaccurate dosing of medications potentially has an impact not only for an individual but also at a public health level.

## Methods/design

### Objectives

We will follow the following steps, modified from the Cochrane approach, for conducting this knowledge synthesis project.

#### Primary objective

What is the bias, precision and accuracy of BSA-corrected eGFR in ml/min/1.73m^2^ (for CKD diagnosis, categorization, prognosis, and decision-making) as compared to measured GFR in the 3 classes of obesity?

#### Secondary objective

What is the bias, precision, and accuracy of BSA-uncorrected eGFR in ml/min (for medication dosing) as compared to measured GFR in the 3 classes of obesity?

We will perform the following sub-group analysis: (1) class of obesity, (2) sex, (3) reference method utilized, (4) categories (stages) of CKD, and (5) race (if data is available).

### Selection criteria

*Participant/population*: For all research questions—adult patients aged ≥ 18 years, with BMI ≥ 30 kg/m^2^ who have had a measured GFR performed.

*Reference standard*: For all research questions—measured GFR by at least one of the reference methods (urinary clearance or radio-isotope clearance of inulin, iothalamate, iohexol, ^51^Cr EDTA [ethylenediaminetetraacetic acid], ^99m^Tc DTPA [diethylene-triamine-pentaacetic acid]).

*Index test*: For all research questions—commonly utilized eGFR equations (see Table 1).

*Target condition*: For all research questions—patients with CKD and BMI ≥ 30 kg/m^2^.

*Studies*: Studies that will meet all of the above criteria will be included in our systematic review.

### Criteria for excluding studies

We will exclude animal studies, studies that were conducted only in patients with age < 18 years, narrative reviews, practice guidelines, and editorials.

#### Step 1: Searching for studies

We will systematically search electronic bibliographic databases including MEDLINE, EMBASE, and the Cochrane Library with no restrictions on language or specific dates. With knowledge user, we have developed a comprehensive search strategy under the help of the medical research librarian. The search terms were adapted for the different databases using a combination of Medical Subject Heading and relevant keywords contained in titles and abstracts. Our preliminary search strategy using Cochrane, MEDLINE, and EMBASE databases have identified 190, 1246, and 1660 citations, respectively (Additional file 1). We will extend our literature searching by (1) reviewing the bibliographic reference lists of studies selected from electronic databases; (2) examining the bibliography of any identified systematic review, narrative review, and opinion for relevant studies; (3) exploring web-based registries of clinical trials (clinicalTrials.gov); (4) using Internet search engines such as Google, Google Scholar, Yahoo!, and Elsevier’s Scirus; and (5) identifying relevant abstracts from professional society meetings including the Canadian Society of Nephrology, American Society of Nephrology and European Renal Association meetings, American Society for Metabolic and Bariatric Surgery, and the Canadian Obesity Network meeting.

#### Step 2: Selecting studies and collecting data

To select studies of interest, at least two authors (SS and AA) will independently screen titles and abstracts of all articles identified and will exclude irrelevant articles. We will calculate the Kappa for agreement between the reviewers. The full-text articles will be requested for included studies of each reviewer after initial screening. These will be reviewed by both the reviewers for the final selection of studies. Disagreement between the two researchers will be resolved by consensus and discussion with a third co-investigator if necessary. A data extraction tool will be developed based on the Cochrane Consumers and Communication Review Group’s data template [29]. Two authors will independently extract and compare the data from the selected studies. If they disagree, the principal investigator (PI) will review the extracted data and assist in making the final decision.

For all studies selected, we will extract information on general study characteristics including the study author, year of data collection, year of publication, country, study design, study participant (age, sex, race, weight, height, BMI, BSA), type of reference standard utilized, protocol of the reference standard administered, the index test studied, the methodology of measurement of index test, causes of CKD, categories of GFR, the proportion of eGFR within 10, 20, 30, 40, and 50% of measured GFR, and bias between eGFR and measured GFR.

#### Step 3: Assessing risk of bias in included studies

To assess the risk of bias, we will use the QUADAS-2 tool [10]. The quality assessment and the presence of potential bias within studies will be assessed independently by the PI (AA) and SS. Specifically for each study, we will assess the quality of methods used to select study population as well as if any bias was introduced because of exclusion of certain patients. We will assess if any potential bias was introduced in the way index test and reference tests were administered. We will assess whether the timing of reference test and index test or flow of patients introduced bias.

#### Step 4: Analyzing data and undertaking meta-analyses

We will review all selected studies. If the quality of methods and risk of bias are adequate, we will perform a meta-analysis. We will assess the heterogeneity using the *χ*^2^ and the *I*^2^ statistics to examine whether the estimates from studies included could be pooled. We will consider *I*^2^ value < 60% as acceptable for pooling results into a meta-analysis. To assess for heterogeneity due to different types of studies that will be included in the systematic review (different reference standard utilized), we will perform subgroup analysis. The overall effects and their 95% CI will be obtained using a random-effects model as described by DerSimonian and Laird [11]. Sensitivity and multivariate meta-regression analyses will be performed to assess the effects of clinical factors and socio-demographic characteristics reported in included studies (i.e., categories of GFR, age, sex, BSA, cause of CKD) on the meta-analytic estimates. If data allows, we will construct hierarchical summary ROC (HSROC) to account for variations at within study level and between studies. All analysis will be performed using the Comprehensive Meta-analysis software (version 2.2.046, Biostat Inc., Englewood, NJ, USA). If it is not possible to conduct meta-analysis because of the heterogeneity of estimates reported by studies, a qualitative narrative synthesis of the evidence will be performed summarizing the key characteristics of studies included, quality of studies, and estimates reported. If the summary of data stratified by different classes of obesity is not available, we will request individual patient data from investigators to perform an individual patient meta-analysis.

#### Step 5: Addressing reporting biases

We will assess publications bias using the funnel plot and the Egger’s statistic will be used to assess for the statistical significance [12].

We will use PRISMA-DTA: Checklist for reporting of diagnostic test accuracy systematic reviews (http://www.equator-network.org/library/reporting-guidelines-under-development/#52).

#### Step 6: Interpreting results and drawing conclusions

At the completion of this systematic review, we will summarize the main findings and discuss the quality of evidence in term of the accuracy of eGFR equations in patients with obesity. In particular, we will address both the category of kidney disease (ml/min/1.73m^2^) and medication dosing (ml/min) stratified by class of obesity. Study limitations, consistency and bias, and plausible confounding factors that would have biased effect and strength of studies will be addressed. Together with the knowledge user and the project experts, we will address the usefulness of the findings for clinical practice and formulate recommendations. A manuscript of the review will be written for publication in an open access journal.

### Timeline

We plan to conduct this project over a 1-year period.

### Challenges of our approaches and other considerations

Given that our review will include data from unpublished studies (i.e., abstracts presented at conferences), it is possible that some of the data available will be insufficient. To overcome this, we will take all efforts to contact the study authors to obtain more complete information from unpublished studies. The second challenge will be the assessment of the accuracy of eGFR where two or more reference standards have been utilized. If data is not available separately in the manuscript, we will contact the authors to provide us that data. The third challenge will be if data is not separately presented for obese patients and not stratified by 3 classes of obesity. Again, we will attempt to obtain that data from authors. We have not included MDRD equation in the index test as it has been superseded by CKD_EPI equation.

## Discussion and potential impact

The most commonly utilized equation in the population for estimating GFR is Chronic Kidney Disease Epidemiology Collaboration (CKD-EPI) creatinine equation [30]. The creatinine-based CKD-EPI equation calculated eGFR is reported by many laboratories automatically, whenever serum creatinine is requested (http://oaml.com/wpcontent/uploads/2016/05/OAMLeGFREPIGuidelineFinal2015.pdf;https://www.calgarylabservices.com/lab-services-guide/lab-tests/AlphabeticalListing/G/Glomerular-Filtration-Rate-Estimated.htm). The equation incorporates age, sex, race, and serum creatinine to calculate eGFR. It gives the results in ml/min/1.73m^2^ (i.e., corrected for a body surface area [BSA] of 1.73m^2^) which is utilized for categorizing (staging) CKD [13]. For medication dosing, this eGFR is supposed to be corrected for the patient’s body surface area (multiplying by 1.73 and dividing by the BSA of the individual) [31]. The CKD-EPI equation was derived from 5504 individuals with a mean BMI of 28 (SD 6) kg/m^2^, with only 29% of patients having diabetes, and was validated in 3875 patients with a mean BMI of 27 (SD 6) kg/m^2^) [29]. Creatinine is a by-product of muscle metabolism; the higher the muscle mass, the higher the serum creatinine at any given level of GFR [32]. In patients with obesity (as compared to non-obese patient with same BSA), the muscle mass may be lower [33]. Thus, this equation might not be best suited for determining eGFR in Canadian population especially in women and in patients with diabetes (which is commonly associated with obesity). In the general population, 80 to 90% of patients have eGFR which are within 30% of reference GFR. *In obese individuals*, *compared to* non-obese individuals with same serum creatinine (and hence same calculated CKD-EPI eGFR), the true GFR may be different than eGFR and accuracy of eGFR may be much more problematic than the general population. MDRD equation [34] has the same variables as CKD-EPI equation. It was published in 1999. It was found to over diagnose CKD and was super seeded by CKD-EPI equation which was published in the year 2009. The bias and precision of MDRD equation was inferior to the CKD-EPI equation [35]. MDRD equation has been replaced by CKD-EPI equation which is more accurate. Thus, we plan not to include MDRD equation in our review. Other formulae have other limitations and factors leading to inaccuracies.

The Cockcroft-Gault formula [36] estimates GFR (creatinine clearance) using serum creatinine, age, weight, and sex. It provides results in milliliters per minute which can be utilized for medication dosing. This formula has extensively been used in pharmacological studies, both historically and in ongoing studies [37–40]. For CKD categories (staging), these should be corrected (standardized) for BSA of 1.73 m^2^ (by multiplying it with BSA of individual and dividing by 1.73) [13]. This equation has weight as a variable which accounts for an individual’s muscle mass. However, in obese individuals, using the actual weight of the individual to calculate the Cockcroft-Gault eGFR would overestimate the muscle mass and hence overestimate the true GFR. Some adjustments in weight are suggested for calculation [33, 36] (such as ideal body weight or adjusted ideal body weight), but are uncommonly done in clinical practice.

Cystatin C is a low molecular weight protein that functions as a cysteine protease inhibitor and is produced at a constant rate by all nucleated cells. It is freely filtered and catabolized in the proximal tubule without being secreted [41]. Serum concentration is inversely related to GFR, and in some studies, it has been shown to be a better marker of GFR than serum creatinine [19, 42, 43]. The most commonly used cystatin C equations are CKD-EPI cystatin C and CKD-EPI creatinine-cystatin [44]. They were developed and internally validated in patients with a mean BMI of 28 [6] kg/m^2^ and externally validated in patients with a mean BMI of 25 [4] kg/m^2^. In the development and internal validation cohort, 31% of patients had a BMI of > 30 kg/m^2^ and external validation cohort had only 13% of patients with BMI > 30 kg/m^2^. Thus, majority of the patients were not obese. In addition, cystatin C concentration is higher in patients with obesity [45], and these equations might not be accurate in obese individuals.

Beta-trace protein (BTP) is a low molecular weight glycoprotein that has been shown to be a more sensitive marker of GFR than creatinine [46] in a number of different patient groups [47–51]. The most commonly used equation for calculation of eGFR by BTP is the CKD-EPI BTP equation [52]. It was developed in patients with mean BMI 30.1 (SD 6.7) kg/m^2^ and internally validated in patients with mean BMI 30.2 (SD 6.7) kg/m^2^. BMI > 30 kg/m^2^ was present in 412 patients and when compared to reference standard, patients with BMI > 30 kg/m^2^ had a significantly higher mean bias. There has been no external validation performed yet of this equation.

This review might help determine the best equation for calculation of GFR in obese individuals for staging of CKD and medication dosing in CKD. It will have a major impact on clinicians who routinely take care of obese individuals with CKD. In addition, if the systematic review finds that current equations are not accurate, this data can be utilized to design a study to develop clinically meaningful equations in this population (class 1, 2, and 3 obesity for both sexes).

## Additional file

Additional file 1: Search Strategy. (PDF 16 kb)

## Data Availability

Not applicable

## Abbreviations

CKD: Chronic kidney disease
CKD-EPI: Chronic Kidney Disease Epidemiology Collaboration
eGFR: Estimated glomerular filtration rate
GFR: Glomerular filtration rate
