# Supplementary material for: Accurate GFR in obesity—protocol for a systematic review
Source: Syst Rev. 2019 Jun 22;8:147. doi: 10.1186/s13643-019-1052-2 (PMC6588895; doi:10.1186/s13643-019-1052-2)
Supplement: Supplementary file 1 — Search Strategy. (PDF 16 kb) [file 13643_2019_1052_MOESM1_ESM.pdf]

Database: Ovid MEDLINE(R) Epub Ahead of Print, In-Process & Other Non-Indexed Citations, Ovid MEDLINE(R) Daily and Ovid MEDLINE(R) <1946 to Present> Search Strategy:

----

1 *Glomerular Filtration Rate/ (8291)
2 estimat*.tw,kw. (898826)
3 1 and 2 (3637)
4 (eGFR or eGFRcr or eGFRcys).tw,kw. (44104)
5 ((gfr or glomerular filtration) adj3 estimat*).tw,kw. (15638)
6 (CKD-EPI or CKDEPI or cysc or cr-cysc or MDRD or Cockcroft-gault or Schwartz or Jelliffe or Wright or Larsson).tw,kw. (10147)
7 "modification of diet in renal disease".tw,kw. (2417)
8 "Chronic Kidney Disease Epidemiology Collaboration".tw,kw. (746)
9 (BTP or beta trace protein).tw,kw. (684)
10 ((cystatin or creatinine) adj4 (equation* or formula* or algorithm*)).tw,kw. (1214)
11 3 or 4 or 5 or 6 or 7 or 8 or 9 or 10 (61168)
12 ((gfr or glomerular filtration) adj3 (measured or measurement)).tw,kw. (2669)
13 mGFR.tw,kw. (237)
14 reference GFR.tw,kw. (64)
15 ((iothalamate or inulin or iohexol or urin*) adj2 clearance*).tw,kw. (4230)
16 plasma disappearance.tw,kw. (909)
17 Technetium Tc 99m Pentetate/ or (Tc-DTPA or Tc-99m-DTPA or 99mTc-DTPA).tw,kw. (3943)
18 (diethylene-triamine-pentaacetat* or "(99m)Tc-diethylenetriamine").tw,kw. (91)
19 (Cr-EDTA or 51Cr-EDTA).tw,kw. (1631)
20 (ethylenediaminetetraacetic acid or ethylenediamine tetraacetic acid).tw,kw. (4768)
21 or/12-20 (16674)
22 11 and 21 (1861)
23 "Sensitivity and Specificity"/ (322527)
24 (sensitivity or specificity).tw,kw. (885133)
25 accur*.tw,kw. (641293)
26 predict*.tw. (1261190)
27 "Predictive Value of Tests"/ (176753)
28 "Reproducibility of Results"/ (344710)
29 (precision or performance).tw,kw. (812041)
30 (reliab* or valid*).tw,kw. (878518)
31 or/23-30 (3809795)
32 22 and 31 (1262)
33 Obesity/ or Obesity, Morbid/ (168456)
34 (obese or obesity or overweight).tw,kw. (256143)
35 Body Surface Area/ or body surface area.tw,kw. (15533)
36 or/33-35 (307831)
37 22 and 36 (175)
38 32 or 37 (1297)
39 animals/ not humans/ (4364700)
40 38 not 39 (1246)

Database: Embase Classic+Embase <1947 to 2017 May 08> Search Strategy:

----

1 estimated glomerular filtration rate/ (3784)
2 (eGFR or eGFRcys or eGFRcr).tw. (73799)
3 ((gfr or glomerular filtration) adj3 estimat*).tw. (23259)
4 (CKD-EPI or CKDEPI or cysc or cr-cysc or MDRD or Cockcroft-gault or Schwartz or Jelliffe or Wright or Larsson).tw. (17883)
5 "modification of diet in renal disease".tw. (3560)
6 "Chronic Kidney Disease Epidemiology Collaboration".tw. (1049)
7 (BTP or beta trace protein).tw. (852)

8 creatinine/ec or cystatin C/ec (95114)
9 ((cystatin or creatinine) adj4 (equation* or formula* or algorithm*)).tw. (1995)
10 or/1-9 (186569)
11 ((gfr or glomerular filtration) adj3 measured).tw. (2995)
12 mGFR.tw. (458)
13 reference GFR.tw. (80)
14 inulin clearance/ (2124)
15 ((iothalamate or inulin or iohexol or urin*) adj2 clearance*).tw. (5837)
16 plasma disappearance.tw. (1212)
17 pentetate technetium tc 99m/ or (Tc-DTPA or Tc-99m-DTPA or 99mTc-DTPA).tw. (6168)
18 (diethylene-triamine-pentaacetat* or "(99m)Tc-diethylenetriamine").tw. (50)
19 (Cr-EDTA or 51Cr-EDTA).tw. (1965)
20 edetate chromium/ or (ethylenediaminetetraacetic acid or ethylenediamine tetraacetic acid).tw. (5106)
21 or/11-20 (21858)
22 10 and 21 (3544)
23 diagnostic accuracy/ or diagnostic value/ or measurement accuracy/ (343255)
24 accur*.tw. (789270)
25 "sensitivity and specificity"/ or (sensitivity or specificity).tw. (1212427)
26 predictive value/ (107948)
27 intermethod comparison/ (219753)
28 (precision or performance).tw. (944189)
29 or/23-28 (2935976)
30 22 and 29 (1508)
31 obesity/ or morbid obesity/ (353934)
32 (obese or obesity or overweight).tw. (355588)
33 body weight/ or body surface/ or body surface area.tw. (280283)
34 or/31-33 (669941)
35 22 and 34 (450)
36 30 or 35 (1717)
37 (animals/ or animal experiment/) not humans/ (3203905)
38 36 not 37 (1660)

Database: EBM Reviews - Cochrane Central Register of Controlled Trials <March 2017> Search Strategy:

----

1 Glomerular Filtration Rate/ (2213)
2 estimat*.tw,kw. (40443)
3 1 and 2 (730)
4 (eGFR or eGFRcr or eGFRcys).tw,kw. (2883)
5 ((gfr or glomerular filtration) adj3 estimat*).tw,kw. (2029)
6 (CKD-EPI or CKDEPI or cysc or cr-cysc or MDRD or Cockcroft-gault or Schwartz or Jelliffe or Wright or Larsson).tw,kw. (798)
7 "modification of diet in renal disease".tw,kw. (291)
8 "Chronic Kidney Disease Epidemiology Collaboration".tw,kw. (73)
9 (BTP or beta trace protein).tw,kw. (41)
10 ((cystatin or creatinine) adj4 (equation* or formula* or algorithm*)).tw,kw. (118)
11 3 or 4 or 5 or 6 or 7 or 8 or 9 or 10 (4589)
12 ((gfr or glomerular filtration) adj3 (measured or measurement)).tw,kw. (393)
13 mGFR.tw,kw. (51)
14 reference GFR.tw,kw. (3)
15 ((iothalamate or inulin or iohexol or urin*) adj2 clearance*).tw,kw. (643)
16 plasma disappearance.tw,kw. (71)
17 Technetium Tc 99m Pentetate/ or (Tc-DTPA or Tc-99m-DTPA or 99mTc-DTPA).tw,kw. (200)

18 (diethylene-triamine-pentaacetat* or "(99m)Tc-diethylenetriamine").tw,kw. (4)
19 (Cr-EDTA or 51Cr-EDTA).tw,kw. (162)
20 (ethylenediaminetetraacetic acid or ethylenediamine tetraacetic acid).tw,kw. (100)
21 or/12-20 (1399)
22 11 and 21 (190)
